# Comparative Evidence on Negative Pressure Therapy and Hyperbaric Oxygen Therapy for Diabetic Foot Ulcers: A Systematic Review of Independent Effectiveness and Clinical Applicability

**DOI:** 10.3390/medicina62010109

**Published:** 2026-01-04

**Authors:** Álvaro Astasio-Picado, Jesús Jurado-Palomo, Belén Pozo-Aranda, Paula Cobos-Moreno

**Affiliations:** 1 Physiotherapy, Nursing and Physiology Department, Faculty of Health Sciences, University of Castilla-La Mancha, 45600 Toledo, Spain; 2 Department of Nursing, University Center of Plasencia, University of Extremadura, 10600 Cáceres, Spain

**Keywords:** diabetic foot ulcer, negative pressure therapy, hyperbaric oxygen therapy, wound healing, amputation prevention

## Abstract

*Background and Objectives*: To evaluate and synthesize evidence on the independent clinical effectiveness, safety, and applicability of Negative Pressure Wound Therapy (NPWT) and Hyperbaric Oxygen Therapy (HBOT) in diabetic foot ulcers (DFUs), and to determine whether current evidence allows for a direct comparison between both interventions: NPWT and HBOT are widely advanced therapies for DFUs. Although both show benefits, the relative superiority of one over the other remains unclear. Systematic review of the literature conducted in accordance with PRISMA guidelines. *Materials and Methods*: A comprehensive literature search was performed using two electronic databases. The review included randomized controlled trials, systematic reviews, meta-analyses, and non-randomized studies. Methodological quality and risk of bias were assessed using validated tools: RoB 2.0 for randomized trials, AMSTAR-2 for systematic reviews, and ROBINS-I for non-randomized studies. *Results*: A total of 22 studies were included. NPT was shown to be effective in accelerating wound healing, though results varied depending on the type of intervention and clinical context. HBOT demonstrated beneficial effects on angiogenesis and significantly reduced the rate of major amputations. Both therapies presented significant advantages in the management of diabetic foot ulcers. *Conclusions*: Negative pressure therapy and hyperbaric oxygen therapy are both effective treatments for diabetic foot ulcer healing. However, treatment selection should be individualized based on patient-specific clinical factors, ulcer severity, and available healthcare resources. Integrating these advanced therapies within a multidisciplinary care approach may optimize outcomes and reduce the risk of complications. Future research should include standardized, head-to-head RCTs.

## 1. Introduction

Diabetic foot (DF) is one of the most common, debilitating, and costly complications of diabetes mellitus (DM), affecting a substantial proportion of patients and representing a major cause of morbidity, mortality, and long-term disability worldwide [1]. Diabetic foot ulcers (DFUs), in particular, constitute a major clinical challenge due to their complex pathophysiology and their high propensity to deteriorate into deep infections, osteomyelitis, or severe ischemic damage, frequently culminating in partial or major lower-limb amputation [2]. This cascade of events profoundly affects patients’ quality of life by reducing functional independence, mobility, and social participation, while simultaneously generating a disproportionate economic burden for healthcare systems and families [3].

DM is a chronic endocrine-metabolic disorder characterized by persistent hyperglycemia resulting from impaired insulin secretion, insulin action, or both [4,5,6,7]. Over recent decades, the global prevalence of DM has risen dramatically, driven by aging populations, sedentary lifestyles, increased rates of obesity, and greater exposure to energy-dense diets [8]. This escalation has been particularly pronounced in low- and middle-income countries, where rapid urbanization and limited access to preventive care have intensified the public health impact of the disease. Among its chronic complications, diabetic foot pathology (PD) stands out as one of the most severe due to its multifactorial nature and the wide spectrum of associated clinical consequences [5,6,7,8,9,10,11,12,13].

DFU development results from the convergence of three major physiopathological processes peripheral neuropathy, peripheral arterial disease, and biomechanical foot alterations. Sensory neuropathy diminishes protective sensation and prevents recognition of repetitive trauma; motor neuropathy leads to structural deformities and abnormal plantar pressures; while autonomic neuropathy reduces sweating, leading to dry, fissured skin vulnerable to injury [5,13,14,15]. In parallel, peripheral arterial disease compromises tissue oxygenation, impeding wound healing, and worsening susceptibility to infection. Once an ulcer appears, impaired immune responses, reduced leukocyte function, and persistent hyperglycemia foster bacterial colonization and chronic inflammation, generating an ideal environment for non-healing wounds.

The magnitude of the problem is alarming: according to the International Diabetes Federation, more than 4 million people develop DFUs every year [1], and up to 25% of individuals with diabetes will experience at least one ulcer during their lifetime. Recurrence is common, with nearly 40% of healed ulcers reappearing within one year and up to 65% within five years. DFUs remain the leading cause of non-traumatic lower-limb amputations globally, with amputation risk up to 20 times higher in diabetic populations. Post-amputation mortality is high, reaching 70% at five years, surpassing many cancer survival rates [14].

Beyond the individual burden, DFUs impose an immense financial challenge. Their management requires intensive healthcare resources—hospital admissions, diagnostic procedures, revascularization surgeries, prolonged antibiotic therapies, advanced wound care, and long-term rehabilitation. According to global estimates, DFU treatment consumes billions of dollars annually, and costs increase exponentially when complications or amputations occur, further underscoring the imperative for early, effective interventions.

Traditional management strategies—consisting of glycemic control, wound debridement, infection treatment, vascular evaluation, antimicrobial therapy, pressure offloading, and moisture-balanced dressings—form the cornerstone of DFU care. However, despite their importance, healing is often slow, and recurrence remains distressingly common [3]. This clinical reality has prompted the integration of advanced adjunctive therapies capable of enhancing the wound microenvironment, promoting perfusion, and accelerating tissue regeneration.

Among these innovative interventions, Hyperbaric Oxygen Therapy (HBOT) and Negative Pressure Wound Therapy (NPWT) are two of the most widely studied and increasingly utilized modalities. HBOT involves administering100% oxygen at pressures than atmospheric levels, achieving a substantial rise in dissolved oxygen within plasma. This hyperoxygenation stimulates angiogenesis, enhances fibroblast proliferation and collagen synthesis, augments leukocyte bactericidal activity, and improves the efficacy of certain antibiotics [16,17,18,19]. Clinical studies have suggested that HBOT may reduce major amputation rates and improve healing trajectories in chronic or ischemic DFUs, particularly when microcirculatory impairment plays a critical role.

NPWT, in contrast, applies controlled subatmospheric pressure to the wound through a sealed dressing connected to a vacuum pump. This mechanism removes excess exudate, decreases bacterial burden, reduces interstitial edema, increases local perfusion, and mechanically stimulates granulation tissue formation [14,20]. NPWT has shown considerable therapeutic value in complex, exudative, or deep wounds especially those unresponsive to conventional treatment modalities.

The implementation of both HBOT and NPWT requires skilled multidisciplinary collaboration. In this context, nursing professionals perform essential functions such as wound assessment, device management, patient education, adherence monitoring, identification of early complications, and integration of care strategies [2]. Their clinical role is central to therapy effectiveness and patient outcomes.

Despite the accumulated evidence supporting both interventions, notable conceptual and methodological gaps persist. Although the literature often debates which therapy may offer superior clinical outcomes in terms of efficacy, safety, and applicability, these dimensions have seldom been explored systematically or in a manner that allows direct comparison. Regarding safety, the available studies report adverse events in a heterogeneous and frequently superficial way. For HBOT, side effects such as barotrauma, middle-ear discomfort, headaches, oxygen toxicity, or transient visual changes are described inconsistently across trials [18], while NPWT-related complications including perilesional maceration, bleeding, necrosis, or localized infection—are documented in case series and observational studies but rarely assessed as primary outcomes [21,22]. This fragmented reporting prevents establishing robust and comparable risk profiles for each therapy.

Similarly, applicability has not been evaluated in depth despite being essential for real-world clinical decision-making. HBOT requires access to specialized hyperbaric chambers, trained technical personnel, and high-cost infrastructure, which limits its availability and feasibility in many health systems [16,17,18]. In contrast, NPWT depends on device accessibility, adherence to equipment protocols, and close monitoring by trained wound-care professionals to minimize complications and ensure treatment continuity, all of which directly influence its logistical and economic viability [14,20]. These contextual factors significantly affect therapeutic selection but remain underrepresented in most studies.

Furthermore, no high-quality studies directly comparing NPWT with HBOT exist. Instead, virtually all available evidence evaluates each intervention independently against standard or conventional wound care [21,22,23,24,25,26,27,28,29,30,31,32,33,34,35,36,37,38,39,40,41,42]. This evidence structure, although useful for determining individual therapeutic benefits, does not permit establishing methodologically valid conclusions regarding their relative superiority or optimal indication according to ulcer type, severity, or patient profile. Consequently, the current scientific literature provides valuable but fragmented insights that clarify the isolated effects of NPWT and HBOT but do not resolve the comparative uncertainties that persist in clinical practice.

Given these gaps, a comprehensive and systematic synthesis of the evidence is essential to guide clinical decision-making. The present systematic review aims to evaluate the independent clinical efficacy of NPWT and HBOT in the management of DFUs, with particular emphasis on their contribution to healing outcomes, complication reduction (including the prevention of amputations), and clinical applicability within multidisciplinary care frameworks.

The overarching objective of this study is to analyze and compare the available evidence on NPWT and HBOT as therapeutic strategies for DFUs, with a focus on optimizing healing outcomes and enhancing quality of life, particularly from the perspective of nursing practice, which plays a pivotal role in the management and success of advanced wound therapies.

## 2. Methodology

This paper constitutes a systematic review of the scientific literature aimed at evaluating the efficacy of two innovative therapies—negative pressure therapy (NPT) and hyperbaric oxygen therapy (HBOT)—in the treatment of diabetic foot ulcers (DFUs). The review was designed following a rigorous protocol and in accordance with the guidelines of the PRISMA 2020 Declaration (Supplementary Materials), ensuring a transparent, reproducible, and exhaustive process for the identification, selection, and analysis of relevant studies [43].

The protocol was previously registered in the PROSPERO international retrospective register of systematic reviews (ID: CRD420251273813).

Study eligibility criteria: Inclusion criteria were randomized clinical trials, non-randomized studies, and meta-analyses, publications in English and Spanish, studies available in full text, and research conducted in human populations. Exclusion criteria included publications prior to 2014, dissertations, case reports, literature reviews, short articles, or other documents with a low level of scientific evidence.

To guide this review, the following research question was formulated, structured according to the PICO model:
ElementDescriptionP (Population)Patients with diabetes mellitus presenting with active diabetic foot ulcers.I (Intervention)NPWT **or** HBOT.C (Comparison)Conventional treatment. *Direct comparison between NPWT and HBOT was not possible due to absence of eligible head-to-head studies*.O (Outcomes)Healing rate, reduction in complications, and improvement in quality of life.

The scientific literature search was conducted in five specialized and widely recognized electronic databases in the biomedical field: PubMed, The Cochrane Library, Web of Science (WOS), SciELO, and Dialnet. These sources were selected for their multidisciplinary coverage and their usefulness in high-quality studies on therapeutic interventions.

A structured search strategy was designed using standardized descriptors in English (MeSH) and Spanish (DeCS), combined using the Boolean operators “AND” and “OR,” to optimize the retrieval of relevant studies. The main search formulas were: “(Diabetic foot ulcer) AND (Negative pressure therapy OR VAC therapy)” and “(Diabetic foot ulcer) AND (Hyperbaric oxygen therapy)”.

In addition, filters were applied to restrict the results to human studies, publications from 2014 to 2025, and languages: English and Spanish.

The selection and data collection process took place between November 2024 and March 2025, according to the previously defined inclusion and exclusion criteria. Article selection was carried out in several stages, including screening by title and abstract: elimination of irrelevant articles; full reading of potentially eligible studies; and final assessment of inclusion based on compliance with the PICO framework.

To facilitate and systematize the process, Rayyan QCRI (web version, 2023) software was used, a support tool for systematic reviews that allows for comparing decisions between reviewers and managing discrepancies.

Included studies had to evaluate the efficacy of NPT or HBOT in patients with active PD ulcers, without restriction by age, sex, or comorbidities, as long as the primary condition was the presence of diabetic ulcers. The outcomes of interest were healing rate, ulcer size reduction, healing time, adverse events or complications, and patient quality of life.

To assess the methodological quality of the included studies, different tools were used depending on the design type:

- RoB 2.0: For randomized controlled trials. It assesses the risk of bias in five critical domains and provides an overall assessment of the risk of bias per outcome (Table A1) [43].
- AMSTAR-2: For meta-analyses and systematic reviews. It analyzes 16 domains and classifies the overall confidence in the quality of the review as high, moderate, low, or critically low (Table A2) [44].
- ROBINS-I: For non-randomized studies. It assesses seven domains, including bias due to confounding, participant selection, intervention classification, and outcomes, among others. Studies are classified as low, moderate, severe, or critical risk (Table A3) [45].

Regarding the evidence synthesis, due to the heterogeneity of the selected studies in terms of design, population, type of intervention, and variables measured, a quantitative meta-analysis was not possible. Instead, a qualitative narrative synthesis was conducted, which groups and analyzes the main findings of the studies, assessing the consistency of the results and their clinical applicability.

Likewise, the GRADE (Grading of Recommendations Assessment, Development and Evaluation) system was applied to evaluate the quality of the evidence and the strength of the recommendations. GRADE classifies evidence into four levels: high, moderate, low, and very low, based on the study design, the consistency of the results, the precision of the estimates, and the risk of bias [46].

## 3. Results

**Figure 1:**
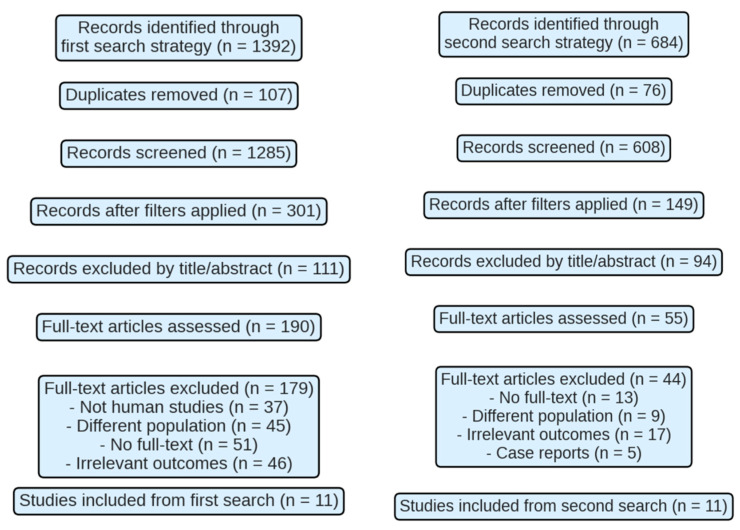
PRISMA diagram of the study selection process.

Study selection process:

1392 records were identified in the first search strategy, of which 107 were eliminated due to duplicate results. After applying filters, 301 studies were screened, excluding 111 after reviewing the title and abstract. A total of 190 full-text articles were evaluated, of which 179 were discarded due to: lack of inclusion of human subjects (*n* = 37), inappropriate population (*n* = 45), inaccessibility of the full text (*n* = 51), or irrelevant results (*n* = 46). Eleven studies were selected (Figure 1).

The second strategy yielded 684 records, of which 76 were duplicates. After applying filters, 149 studies were screened, 94 of which were excluded. Fifty-five full texts were evaluated, with 44 excluded due to text inaccessibility (*n* = 13), inappropriate population (*n* = 9), irrelevant results (*n* = 17), or inadequate design (*n* = 5). Ultimately, 11 studies were included.

A total of 22 studies met the inclusion criteria (Table A4).

General characteristics of the included studies:

The selected studies included 9 meta-analyses, 1 systematic review, 8 randomized controlled trials (RCTs), and 4 non-randomized studies. The geographic distribution was diverse, with China (*n* = 6) and Spain (*n* = 3) being the most represented countries. The sample size ranged from 28 to 1764 participants.

Importantly, none of the included studies directly compared Negative Pressure Wound Therapy (NPWT) with Hyperbaric Oxygen Therapy (HBOT). Instead, all identified investigations evaluated each therapy independently against conventional or standard DFU management strategies. Therefore, the evidence base allows assessment of the *individual* effectiveness of NPWT and HBOT, but does not permit a direct comparative analysis between the two modalities.

For this reason, in the context of the present review, NPWT and HBOT are examined separately in relation to conventional treatment, rather than against each other. This distinction is essential to correctly interpret the findings and to avoid overestimating comparative conclusions that the available evidence cannot support.

Summary of results:

Meta-analyses on NPWT reported improvements in wound healing and tissue quality [23,24,25], while those focusing on HBOT showed reductions in major amputations and improvements in DFU healing in patients with peripheral arterial disease [26,27,28,29,30,31].

In RCTs, NPWT showed additional benefits when combined with antibiotic cement [33], as well as superiority over alginate dressings in terms of perfusion and grafting [34]. However, some studies found no significant differences compared to conventional treatments [35,36]. HBOT, on the other hand, was associated with a higher healing rate, fewer amputations, and improved tissue oxygenation [37,38,39,40].

Non-randomized studies supported the efficacy of NPWT in reducing lesion size and epithelialization [21,22,41], although complications such as perilesional maceration were also reported [21]. An additional study observed a 43.3% wound reduction after four weeks of low-pressure NPWT [42].

The included systematic review [32] showed benefits in transcutaneous oxygenation and ulcer area reduction with HBOT, although without a significant impact on amputation rates.

**Figure 2:**
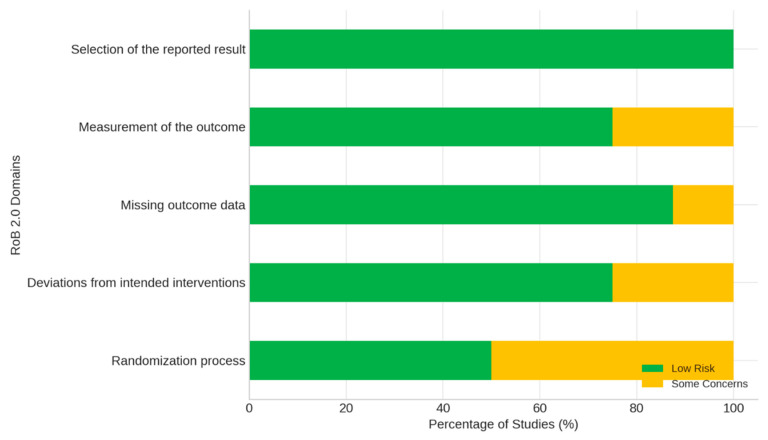
Risk of Bias in Randomized Controlled Trials (RoB 2.0).

Risk of bias assessment:

RoB 2.0 was used for RCTs, AMSTAR-2 for reviews and meta-analyses, and ROBINS-I for non-randomized studies (Figure 2).

In the RCTs, 75% showed low overall risk, although concerns were noted regarding randomization (50%) and outcome measurement (25%).

In the meta-analyses and reviews, 80% showed high confidence, although 40% did not disclose funding sources and 60% did not adequately include PICO components.

The non-randomized studies showed moderate overall risk, primarily due to bias in outcome measurement.

**Figure 3:**
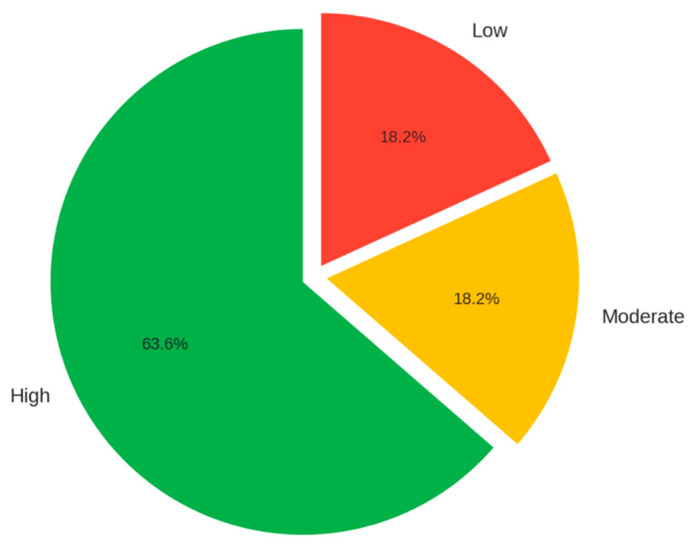
Certainty of Evidence According to GRADE.

Certainty of evidence:

According to the GRADE tool, 14 studies were classified as high quality, 4 as moderate, and 4 as low. The high-quality studies showed low risk of bias and consistent results. Methodological limitations in moderate- or low-quality studies included the presence of bias, low precision, or uncontrolled confounding, which affect confidence in their results (Figure 3).

## 4. Discussion

Diabetic foot ulcers (DFUs) are one of the most serious and frequent complications in people with diabetes, associated with high morbidity, prolonged hospitalization, decreased quality of life, and increased risk of lower-limb amputation. The chronicity and multifactorial origin of DFUs—often involving infection, ischemia, and neuropathy—require comprehensive and individualized treatment strategies. In recent years, advanced therapies such as Negative Pressure Wound Therapy (NPWT) and Hyperbaric Oxygen Therapy (HBOT) have gained relevance for their potential in promoting faster and more effective healing [13,14,15,16,17].

Effectiveness of Negative Pressure Wound Therapy (NPWT)

NPWT has been extensively studied and is considered one of the most effective approaches to promote healing in complex diabetic ulcers. Studies such as those by Chen L et al. [23] and Lavery LA et al. [42] demonstrated significant benefits in terms of wound area reduction, accelerated granulation tissue formation, and overall faster healing compared to conventional treatment. Similarly, Dalmedico MM et al. [24] and Wu Y et al. [34] reported higher graft survival and reduced neutrophil extracellular trap formation, respectively, when NPWT was applied in DFUs, suggesting additional biological benefits that extend beyond simple mechanical effects.

NPWT has also shown positive results from the perspective of both patients and healthcare providers. For example, Palomar Llatas F et al. [41] used the FEDPALLA and VAS scales to assess the impact of NPWT on comfort and quality of care, concluding that this therapy improved patients’ perception of treatment while optimizing clinical management.

Despite these advantages, the evidence is not universally consistent. Studies by Seidel D et al. [35], Liu Z et al. [25], and Lavery LA et al. [36] did not find statistically significant differences in healing rates when comparing NPWT to standard care, nor when comparing different NPWT modalities (e.g., high vs. low pressure), suggesting that its success may depend on other factors such as wound classification, duration of diabetes, or infection status.

The combination of NPWT with other treatment modalities appears to enhance its effectiveness. For instance, Zhong M et al. [33] observed that combining NPWT with antibiotic-loaded bone cement improved the wound bed quality by reducing inflammation (notably via IL-6 modulation) and promoting a favorable M1/M2 macrophage balance, both of which are essential for tissue regeneration.

However, complications linked to NPWT have also been reported. García Oreja S et al. [21] and Lázaro Martínez JL et al. [22] documented adverse events such as perilesional maceration, bleeding, necrosis, and localized infection. Although most patients still experienced satisfactory wound healing, these complications highlight the importance of close monitoring during therapy.

Effectiveness of Hyperbaric Oxygen Therapy (HBOT)

HBOT has also been studied as a complementary treatment for DFUs, especially in cases where chronic ischemia and tissue hypoxia hinder healing. By delivering 100% oxygen at elevated atmospheric pressures, HBOT increases tissue oxygen tension, promoting angiogenesis, fibroblast proliferation, and enhanced antimicrobial activity [32,40].

The study by Nik Hisamuddin NAR et al. [40] supports these mechanisms, showing that HBOT combined with conventional wound care (debridement, irrigation, and dressings) significantly reduced wound size and improved markers of tissue oxygenation. These findings are reinforced by systematic reviews and clinical trials conducted by Elraiyah T et al. [26], Sharma R et al. [30], Chen CY et al. [37], and Kumar A et al. [39], who observed enhanced healing rates and a notable reduction in major amputations, a key outcome in DFU treatment.

In comparative studies, HBOT also outperformed other advanced therapies. A study comparing HBOT to low-level laser therapy showed a significantly greater reduction in wound area with HBOT (89.76% vs. 89.5%), although combining both therapies did not yield additional benefits [38]. This suggests that while HBOT is effective, synergistic approaches may not always result in improved outcomes and must be evaluated on a case-by-case basis.

Nonetheless, HBOT is not without risks. Adverse events such as barotrauma, tinnitus, headaches, and episodes of hypoglycemia have been reported [27,28,29,30,31,32]. While most are mild and self-limiting, careful patient selection and monitoring are crucial, especially in those with pre-existing pulmonary, cardiovascular, or neurologic conditions.

Comparative Analysis and Clinical Considerations

Both NPWT and HBOT offer distinct advantages in the management of DFUs. NPWT is especially effective in reducing wound size, promoting granulation tissue, and decreasing local edema. It is particularly suitable for deep, exudative wounds with infection risk. On the other hand, HBOT is more appropriate for ischemic or refractory wounds where hypoxia plays a central role, and its systemic effects on oxygenation and angiogenesis may benefit tissues beyond the ulcer site.

However, both treatments also present limitations. NPWT may be less effective in ischemic wounds or in cases of poor glycemic control, and it carries a non-negligible risk of local complications [21,22]. Meanwhile, HBOT, despite its systemic benefits, involves higher costs, specialized infrastructure, and risks related to oxygen toxicity [30]. Importantly, neither therapy is a panacea and should be integrated into a multidisciplinary care plan that includes glycemic control, infection management, and pressure offloading [47,48,49].

Limitations and Future Research

The current body of evidence is limited by the heterogeneity of study designs, small sample sizes, variability in outcome measures, and short follow-up durations. In particular, differences in NPWT pressure settings and HBOT treatment protocols make comparisons difficult and hinder the generalizability of results.

- Future studies should focus on:
- Standardizing treatment protocols for both NPWT and HBOT.
- Conducting large-scale randomized controlled trials with long-term follow-up.
- Evaluating cost-effectiveness and quality-of-life outcomes.
- Exploring the molecular and immunologic mechanisms behind treatment efficacy.
- Investigating combination therapies and personalized approaches based on wound etiology and patient profile.

## 5. Conclusions

Based on the available evidence, this systematic review indicates that both Negative Pressure Wound Therapy (NPWT) and Hyperbaric Oxygen Therapy (HBOT) demonstrate **independent clinical benefits** in the management of diabetic foot ulcers (DFUs). However, the heterogeneity of study designs, populations, and outcome measures—and the absence of direct comparative studies—prevents establishing whether one therapy is superior to the other.

NPWT shows consistent improvements in wound granulation and ulcer size reduction when compared with conventional treatment, although the magnitude of these effects varies across studies and patient characteristics. HBOT demonstrates benefits particularly in chronic or ischemic DFUs, including enhanced tissue oxygenation and a reduction in major amputations, though its use is limited by safety considerations, cost, and the need for specialized infrastructure.

Given that no included study directly compared NPWT and HBOT, **this review cannot determine comparative efficacy between the two interventions**. Therefore, treatment decisions should rely on the individual clinical profile of the patient, ulcer etiology, resource availability, and multidisciplinary assessment.

Nursing professionals play a crucial role in implementing both therapies, ensuring appropriate monitoring, patient education, and early detection of complications, thereby contributing to improved healing outcomes and overall quality of care.

## Data Availability

Not applicable. No new data were created.

## Supplementary Materials

The following supporting information can be downloaded at: https://www.mdpi.com/article/10.3390/medicina62010109/s1, PRISMA 2020 Checklist [50].

## Appendix A

**Table A1:** Assessment of risk of bias using the RoB 2 scale (1 (Low risk of bias); 2 (Moderate risk of bias); 3 (High risk of bias)).

	Zhong M et al. [33]	Wu Y et al. [34]	Seidel D et al. [35]	Lavery LA et al. [36]	Chen CY et al. [37]	El-Deen HAB et al. [38]	Kumar A et al. [39]	Nik Hisamuddin NAR et al. [40]
D1: Bias in the randomization process	1	1	1	1	1	1	1	1
D2: Bias due to deviations from the intended interventions	1	1	1	1	1	1	1	1
D3: Bias due to missing outcome data	1	1	1	1	1	1	1	1
D4: Bias in outcome measurement	1	1	1	1	1	1	1	1
D5: Bias in the selection of the reported outcome	1	1	1	1	1	1	1	1
Overall assessment	1	1	1	1	1	1	1	1

**Table A2:** Assessment of risk of bias using the AMSTAR-2 scale (1 (yes); 2 (no)).

	Chen L et al. [23]	Dalmedico MM et al. [24]	Liu Z et al. [25]	Elraiyah T et al. [26]	Brouwer RJ et al. [27]	Chen HR et al. [28]	OuYang H et al. [29]	Sharma R et al. [30]	Kranke P et al. [32]	Moreira DA Cruz DL et al. [31]
D1: PICO Question	2	1	2	2	1	2	1	1	2	2
D2: Pre-established Method	1	1	1	1	1	1	1	1	1	1
D3: Justification of Study Designs	1	1	1	1	1	1	1	1	1	1
D4: Search Strategy	1	1	1	1	1	1	1	1	1	1
D5: Selection of Studies in Duplicate	1	1	1	1	1	1	1	1	1	1
D6: Data Extraction in Duplicate	2	1	1	1	1	1	1	1	1	1
D7: List and Justification of Excluded Studies	1	1	1	1	1	1	1	1	1	1
D8: Description of Included Studies	1	1	1	1	1	1	1	1	1	1
D9: Assessment of Risk of Bias	1	1	1	1	1	1	1	1	1	1
D 10: Sources of Funding for Studies	2	1	1	1	1	2	2	2	1	1
D11: Statistical Methods in Meta-Analysis	1	1	1	1	1	1	1	1	1	1
D12: Impact of Bias in Meta-Analysis	1	1	1	1	1	1	1	1	1	1
D13: Consideration of Bias in Interpretation	1	1	1	1	1	1	1	1	1	1
D14: Explanation of Heterogeneity	1	1	1	1	1	1	1	1	1	1
D15: Research on Publication Sessions	1	1	1	1	1	1	1	1	1	1
D16: Conflict of Interest and Funding	1	1	1	1	1	1	1	1	1	1
Confidence	Medium	High	High	High	High	Medium	High	High	High	High

**Table A3:** Assessment of risk of bias using the ROBINS-I scale (1 (Low risk of bias); 2 (Moderate risk of bias); 3 (High risk of bias)).

	García-Oreja S et al. [21]	Lázaro-Martínez JL et al. [22]	Palomar-Llatas F et al. [41]	Lavery LA et al. [42]
D1: Bias due to confounding factors	2	1	1	1
D2: Bias due to participant selection	1	1	1	1
D3: Bias in the classification of interventions	1	1	1	1
D4: Bias due to deviations from the planned interventions	1	1	1	1
D5: Bias due to missing data	2	1	1	1
D6: Bias in the measurement of results	2	2	2	2
D7: Bias in the selection of the reported outcome	1	1	1	1
Overall assessment	2	2	2	2

**Table A4:** Summary table of the studies included in the review.

Author/Year	Objective	Key Results	Conclusions
Chen L, Zhang S, Da J, Wu W, Ma F, Tang C, Li G, Zhong D, and Liao B/2021 [23]	The aim of this study was to evaluate the efficacy and safety of negative pressure wound therapy (NPWT) in the treatment of diabetic foot ulcers (DF).	Data were collected from 9 randomized controlled trials (943 patients) comparing NPWT (intervention group) with conventional wound care (control group) in patients with DF.	NPWT significantly accelerates wound healing and reduces the time to granulation tissue formation compared with conventional treatment, without increasing the incidence of adverse events or the amputation rate.
Zhong M, Guo J, Qahar M, Huang G y Wu J/2024 [33]	The aim of this study was to analyze the efficacy and underlying mechanisms of negative pressure wound therapy (NPWT) and antibiotic-loaded bone cement (ALBC) in the treatment of diabetic foot ulcers.	In this trial, 28 patients were randomly assigned to two groups: one received NPWT alone (16 individuals) and the other received NPWT combined with ALBC (12 patients).	The group treated with NPWT and ALBC showed a better quality wound bed, with less inflammation, higher collagen quality, and improved vascularization compared to the group that received NPWT alone. In addition, the results indicated a reduction in IL-6 levels and an increase in the expression of healing-related markers.
García-Oreja S, Navarro-González Moncayo J, Sanz-Corbalán I, García-Morales EA, Álvaro-Afonso FJ, and Lázaro-Martínez JL/2017 [21]	The objective of this study was to evaluate the complications associated with negative pressure wound therapy (NPWT) in the treatment of diabetic foot ulcers. Retrospective observational study.	In this study, 57 patients with diabetic foot ulcers were included. They were treated with surgical debridement and subsequently received NPWT at a pressure of 125 mmHg for 7 to 10 days.	Regarding the results, 48 patients experienced some complication, with perilesional maceration being the most common (49%), followed by bleeding (14%), necrosis (12%), local infection (7%), and pain (2%). Only 9 patients (16%) did not experience any complications. Despite this, 80% of patients had favorable results after treatment.
Lázaro-Martínez JL, García-Morales E, Aragón-Sánchez J, García-Álvarez Y, Matilla AC, and Álvaro-Afonso FJ/2014 [22]	The objective of this study was to evaluate the effectiveness of negative pressure wound therapy (NPWT) in the postoperative management of severe infections in diabetic foot ulcers.	In After surgical debridement, NPWT was applied to control infection and bleeding. Subsequently, dressings were changed every 48–72 h, assessing the need to continue therapy. Once bone coverage and complete granulation were achieved, moist wound healing was continued.	Regarding the results, of the 42 patients treated, 38 (90.5%) achieved healing after using VAC^®^ therapy, with a mean healing time of 13 weeks. Patients with neuropathic ulcers healed in 13.38 ± 8.77 weeks, while those with neuroischemic ulcers healed in 16.69 ± 8.84 weeks. 9.5% (4 patients) were referred to the hospital due to serious complications.
Dalmedico MM, do Rocio Fedalto A, Martins WA, de Carvalho CKL, Fernandes BL e Ioshii SO/2024 [24]	The aim of this study was to analyze the effectiveness of negative pressure wound therapy (NPWT) in the management of diabetic foot ulcers compared to different types of dressings or placebo.	Fourteen randomized controlled trials were analyzed in which NPWT was applied to patients with diabetic foot ulcers using variable pressures, comparing the results with conventional treatments, such as moist dressings or bovine collagen.	The results showed a higher healing rate and a more significant reduction in wound area with NPWT compared to conventional treatments in most cases. Three studies indicated a lower incidence of amputations in the NPWT group, although one did not find a significant difference. Heterogeneity in the studies and variations in the applied pressure limited the generalizability of the results.
Palomar-Llatas F, Fornes-Pujalte B, Sierra-Talamantes C, Murillo-Escutia A, Moreno-Hernández A, Díez-Fornes P, Palomar-Fons R, Torregrosa-Valles J, Debón-Vicent L, Marín-Bertolín S, Carballeira-Braña A, Guerrero-Baena/2015 [41]	The objective of this study was to evaluate the efficacy of topical negative pressure wound therapy (NPWT) in the healing of acute wounds, diabetic foot ulcers, venous ulcers, and pressure ulcers.	The study included a sample of 57 patients who received topical NPWT. The FEDPALLA and EVA scales were used to assess the lesions, and a planimetric and dimensional analysis of each lesion was performed.	Regarding the results, all cases treated with topical TPN showed a considerable reduction in lesion size, as well as improved preparation of the wound bed for epithelialization. This intervention proved beneficial both for patients, by providing them with comfort, and for nursing professionals, by improving the efficiency of direct care management.
Wu Y, Shen G, and Hao C/2023 [34]	The aim of this study was to compare the efficacy of NPWT and alginate dressings in wound bed preparation prior to skin grafting surgery in patients with chronic diabetic foot ulcers.	In this trial, 103 patients were assigned to two groups: the NPWT group (*n* = 52) and the control group (alginate dressings, *n* = 51). Both groups received treatment until healthy granulation tissue was achieved in the wound bed at the time of grafting. Fifty patients in each group were analyzed at the end of the trial.	The group treated with TPN showed shorter surgery time, higher graft survival rates, better blood perfusion, less neutrophil extracellular trap formation, and macrophage polarization towards the M2 phenotype, compared to the control group.
Seidel D, Storck M, Lawall H, Wozniak G, Mauckner P, Hochlenert D, Wetzel-Roth W, Sondern K, Hahn M, Rothenaicher G, Krönert T, Zink K, and Neugebauer E/2020 [35]	The aim of this study was to evaluate the efficacy and safety of negative pressure wound therapy (NPWT) in patients with diabetic foot ulcers by comparing this therapy with standard wound care.	In this trial, 368 patients were randomly assigned in a 1:1 ratio, considering the study site and the severity of the ulcer.	Regarding the results, no significant differences were observed in the wound closure rate or time to closure between the two groups. The wound closure rate was low in both groups.
Liu Z, Dumville JC, Hinchliffe RJ, Cullum N, Game F, Stubbs N, Sweeting M, and Peinemann F/2018 [25]	The aim of this study was to evaluate the effects of negative pressure wound therapy (NPWT) on foot ulcers in people with diabetes mellitus (PD), compared with standard treatment or alternative therapies.	This meta-analysis included 11 randomized controlled trials with 972 participants. Most studies compared NPWT with bandages for the treatment of ulcers in PD, except for one that compared low-pressure NPWT with high-pressure NPWT.	Regarding the results, low-certainty evidence suggests that NPWT may increase the proportion of healed wounds and reduce the healing time of ulcers and postoperative wounds in PD. Furthermore, it has not been determined with certainty whether there are differences between low- and high-pressure NPWT in terms of the number of closed wounds or the occurrence of adverse events.
Lavery LA, Murdoch DP, Kim PJ, Fontaine JL, Thakral G, and Davis KE/2014 [42]	The objective of this study was to analyze the effect of low-pressure (80 mmHg) negative pressure wound therapy (NPWT) on wound area reduction in diabetic foot ulcers.	In this study, 30 patients with foot ulcers were treated with low-pressure NPWT for up to 5 weeks.	Regarding the results, 43.3% of the patients achieved at least a 50% reduction in wound area after 4 weeks of treatment.
Lavery LA, La Fontaine J, Thakral G, Kim PJ, Bhavan K, and Davis KE/2014 [36]	The objective of this study was to compare two negative-pressure wound therapy (NPWT) methods for wounds: one with 125 mmHg pressure and a polyurethane foam dressing, and the other with 75 mmHg pressure and a silicone-coated dressing	In this trial, 40 patients with diabetic foot wounds were randomly assigned to receive NPWT with two different approaches: 75 mmHg with a silicone dressing or 125 mmHg with a polyurethane foam dressing, for 4 weeks or until surgical closure.	Regarding the results, no significant differences were found between the two groups in terms of wound healing. Although the proportions varied slightly, the percentage of surgically closed wounds, with a 50% reduction in area or volume, was similar between the 75 mmHg group and the 125 mmHg group.
Elraiyah T, Tsapas A, Prutsky G, Domecq JP, Hasan R, Firwana B, Nabhan M, Prokop L, Hingorani A, Claus PL, Steinkraus LW, and Murad MH/2016 [26]	The objective of this study was to evaluate the effectiveness of hyperbaric oxygen therapy (HBOT), arterial pump devices, and pharmacological agents, such as pentoxifylline, cilostazol, and iloprost, in the treatment of diabetic foot ulcers.	In this meta-analysis, 18 interventional studies were analyzed, including 9 randomized controlled trials with a total of 1526 patients.	Regarding the results, HBOT combined with conventional treatment showed a higher healing rate and a reduction in major amputations compared to standard therapy. Arterial pump devices promoted healing in small trials, while neither iloprost nor pentoxifylline had a significant impact on the amputation rate.
Brouwer RJ, Lalieu RC, Hoencamp R, van Hulst RA, and Ubbink DT/2020 [27]	The objective of this study was to evaluate the effectiveness of hyperbaric oxygen therapy (HBOT) as a treatment for patients with diabetic foot ulcers and peripheral arterial occlusive disease, particularly in reducing major amputations and other clinical outcomes.	In this meta-analysis, 11 studies (729 patients) were analyzed, including 7 randomized controlled trials, 2 controlled clinical trials, and 2 retrospective cohort studies; 4 were used for quantitative synthesis. Most studies applied a 90 min HBOT protocol, with pressures between 2.2 and 2.8 atmospheres absolute (ATA), administered 5 times per week, with a range of 20 to 60 sessions.	Regarding the results, HBOT reduced major amputations, but there were no significant differences in minor amputations, mortality, or healing time.
Chen CY, Wu RW, Hsu MC, Hsieh CJ, and Chou MC/2017 [37]	The aim of this study was to compare the effect of standard care in combination with hyperbaric oxygen therapy (HBOT) versus standard care alone.	This was a randomized controlled trial. In this trial, 38 patients were randomly assigned to two groups: an experimental group (HBOT plus conventional treatment) and a control group (standard care). The experimental group received HBOT in a hyperbaric chamber for 120 minutes at 2.5 ATA, 5 days a week for 4 weeks. Standard care consisted of nutritional management, debridement, topical therapy, and medication.	Regarding the results, the experimental group achieved a total healing rate of 25% compared to 5.5% in the control group; the amputation rate was 5%, while the standard care group had an amputation rate of 11%. Finally, the TOHB group showed considerable improvements in terms of inflammation indices, blood flow and quality of life, with a significant decrease in HbA1c observed.
Chen HR, Lu SJ, Wang Q, Li ML, Chen XC, and Pan BY/2024 [28]	The objective of this study was to assess the efficacy and safety of hyperbaric oxygen therapy (HBOT) as a treatment for diabetic foot ulcers.	This meta-analysis included 29 randomized controlled trials with a total of 1764 patients divided into two groups: one received HBOT (877 patients) and the other conventional treatment (887 patients).	Regarding the results, the HBOT group showed a considerable increase in the complete ulcer healing rate (46.76% vs. 24.46% with conventional treatment). A decrease in the amputation rate was also observed, but with an increase in the incidence of adverse effects (17.37% vs. 8.27%).
El-Deen HAB, Wadee AN, Eraky ZS, Elmasry HM, El-Sayed MS, and Fahmy SM/2023 [38]	The objective of this study was to compare the efficacy of low-intensity laser therapy and hyperbaric oxygen therapy (HBOT) for treating chronic diabetic foot ulcers.	In this randomized controlled trial, 100 patients were randomly assigned to four groups: a) a control group (standard treatment), b) a low-intensity laser therapy group (3 times per week), c) an HBOT group (5 times per week), and d) a combined group (HBOT and low-intensity laser therapy), all for six weeks.	Regarding the results, the groups treated with low-intensity laser therapy, HBOT, and the combined group showed significant improvements in both ulcer area and volume reduction, unlike the control group, which showed no change. The hyperbaric oxygen therapy (HBOT) group achieved the greatest reduction in ulcer area, at 89.76%, while the laser treatment stood out with an 89.5% reduction in volume. The combination of both therapies proved no more beneficial than applying each one individually.
OuYang H, Yang J, Wan H, Huang J, Yin Y/2024 [29]	The aim of this study is to provide physicians with information on the risks and benefits of different treatment options when choosing different treatment strategies for UPD.	Fifty-seven RCTs involving 4826 patients with diabetic foot ulcers were included. Compared with standard of care (SOC), several interventions—particularly platelet-rich plasma (PRP), hyperbaric oxygen therapy (HBOT), topical oxygen therapy (TOT), acellular dermal matrix (ADM), and stem cells—significantly improved complete ulcer healing rates in both direct and network meta-analyses. Combined therapies, especially PRP plus negative pressure wound therapy (NPWT), showed superior efficacy over NPWT alone. No significant differences were observed among interventions in ulcer area reduction. PRP and NPWT shortened healing time compared with SOC, with the greatest benefit observed for PRP combined with ultrasonic debridement and NPWT. PRP was also associated with reduced amputation rates and fewer adverse events. Overall ranking favored combination therapies (PRP + NPWT and UD + NPWT), while SOC consistently showed the poorest outcomes.	Due to the particularity of the wound of DFU, the standard of care is not effective, but the new treatment scheme has a remarkable effect in many aspects. And the treatment of DFU is not a single choice, combined with a variety of methods often achieve better efficacy, and will not bring more adverse reactions.
Sharma R, Sharma SK, Mudgal SK, Jelly P, and Thakur K/2021 [30]	The objective of this study was to examine the efficacy of hyperbaric oxygen therapy (HBOT) as adjunctive treatment for diabetic foot ulcers, comparing its impact on complete ulcer healing and reduction in the amputation rate with standard treatment.	This meta-analysis analyzed 14 studies (12 randomized controlled trials and 2 controlled clinical trials) with a total of 768 participants (384 in the HBOT group and 384 in the standard treatment control group). Standard treatment consisted of glycemic control, antibiotics, and surgical debridement.	Regarding the results, HBOT showed a higher healing rate compared with standard treatment. A considerable reduction in major amputations was observed in the hyperbaric oxygen therapy (HBOT) group, while there were no significant differences in minor amputations or the overall amputation rate. However, patients treated with HBOT experienced more adverse events, such as oxygen toxicity, hypoglycemia, cataracts, and barotrauma. No significant differences were observed in mortality or wound area reduction.
Kumar A, Shukla U, Prabhakar T, Srivastava D/2020 [39]	The aim of this study was to evaluate the efficacy of hyperbaric oxygen therapy (HBOT) as an adjunct to standard therapy and to compare it with standard therapy alone in terms of diabetic foot ulcer healing.	Randomized controlled trial. In this trial, 60 patients were randomly assigned to two groups: group H, which received standard treatment plus HBOT, and group S, which received only standard treatment (glucose control, dressing changes, debridement, and infection control). HBOT was administered at a pressure of 2.4 ATA for 90 minutes, with 6 sessions.	Regarding the results, in group H, 78% of patients healed completely without surgical intervention, while in group S, no patients healed without surgery. Healing time was considerably shorter in group H. Regarding amputations, 7% of individuals in group H required distal amputation, while 11% in group S required proximal amputation. No major amputation was observed in group H.
Kranke P, Bennett MH, Martyn-St James M, Schnabel A, Debus SE, Weibel S/2015 [32]	The objective of this study was to analyze the benefits and risks of hyperbaric oxygen therapy (HBOT) in the healing of chronic lower limb ulcers and its impact on amputation reduction.	In this systematic review, 12 randomized clinical trials with 577 patients were analyzed; 281 received HBOT and 267 received control treatment.	Regarding the results, HBOT did not show a statistically significant reduction in the major and minor amputation rates. Several studies reported a significant increase in transcutaneous oxygenation in the HBOT group. A significant reduction in ulcer area and an improvement in quality of life, particularly in physical function and mental health, were also observed. Some studies reported minor adverse effects, such as ear barotrauma, claustrophobia, tinnitus, and headache, but no serious complications were reported.
Moreira DA Cruz DL, Oliveira-Pinto J, and Mansilha A/2022 [31]	The objective of this study was to evaluate the effectiveness of hyperbaric oxygen therapy (HBOT) for the treatment of diabetic foot ulcers compared to conventional treatment.	This meta-analysis included a total of 13 randomized controlled trials comparing two groups of patients with ulcers: a conventional treatment group (glycemic control, wound debridement, dressings, pressure offloading, and antibiotic use) and a HBOT group (combination of standard treatment and oxygen therapy).	Regarding the results, patients treated with HBOT showed a lower rate of major amputations; however, no significant differences were found between the groups in minor and total amputations. Compared to standard treatment, HBOT quadrupled the chances of complete ulcer healing, significantly reducing ulcer size within two weeks.
Nik Hisamuddin NAR, Wan Mohd Zahiruddin WN, Mohd Yazid B, and Rahmah S/2019 [40]	The objective of this study was to determine the effects of HBOT on the healing of diabetic foot ulcers compared to conventional treatment.	This was a randomized controlled trial. Sixty-two patients with diabetes mellitus (DM) and foot ulcers were included and randomly assigned to two groups: an intervention group, which received conventional treatment and HBOT at 2.4 ATA, 90 minutes per session, 5 days a week for 30 sessions; and a control group, which received only conventional treatment.	The intervention group treated with HBOT showed a significantly greater reduction in wound size compared to the control group. Furthermore, HBOT improved tissue oxygenation, promoted angiogenesis and reduced inflammation, favoring faster healing.
